# Reducing Training Data Using Pre-Trained Foundation Models: A Case Study on Traffic Sign Segmentation Using the Segment Anything Model

**DOI:** 10.3390/jimaging10090220

**Published:** 2024-09-07

**Authors:** Sofia Henninger, Maximilian Kellner, Benedikt Rombach, Alexander Reiterer

**Affiliations:** 1 Fraunhofer Institute for Physical Measurement Techniques IPM, 79110 Freiburg, Germany; maximilian.kellner@ipm.fraunhofer.de (M.K.); benedikt.rombach@ipm.fraunhofer.de (B.R.); alexander.reiterer@ipm.fraunhofer.de (A.R.); 2 Department of Sustainable Systems Engineering INATECH, Albert Ludwigs University Freiburg, 79110 Freiburg, Germany

**Keywords:** semantic segmentation, segment anything model, Mask R-CNN, training data reduction, traffic signs

## Abstract

The utilization of robust, pre-trained foundation models enables simple adaptation to specific ongoing tasks. In particular, the recently developed Segment Anything Model (SAM) has demonstrated impressive results in the context of semantic segmentation. Recognizing that data collection is generally time-consuming and costly, this research aims to determine whether the use of these foundation models can reduce the need for training data. To assess the models’ behavior under conditions of reduced training data, five test datasets for semantic segmentation will be utilized. This study will concentrate on traffic sign segmentation to analyze the results in comparison to Mask R-CNN: the field’s leading model. The findings indicate that SAM does not surpass the leading model for this specific task, regardless of the quantity of training data. Nevertheless, a knowledge-distilled student architecture derived from SAM exhibits no reduction in accuracy when trained on data that have been reduced by 95%.

## 1. Introduction

Traffic signs are an essential component of the infrastructure that enables the safe movement of all road users, including drivers, passengers, cyclists, and pedestrians. They provide unambiguous instructions and warnings that facilitate the prevention of accidents and reduce the risk of injuries or fatalities. It should be noted, however, that this only applies to traffic signs that are intact, correctly oriented, and undamaged. Due to the large number of traffic signs, manually inspecting, localizing, and constantly monitoring them is almost impossible. In Germany alone, for instance, the official traffic sign catalog lists a total of 1134 different traffic signs [1]. These include danger signs, regulatory signs, and direction signs, which are further complemented by traffic devices and additional signs. The estimated number of traffic signs on German roads is approximately 20 million [2]. This equates to one sign every 24 m along the entire street network of 830,000 km [3].

The automatic detection and segmentation of traffic signs has the potential to enhance the efficiency of the process of ensuring correctness. To solve these vision tasks, deep learning is the current state-of-the-art approach [4]. Traditionally, training robust models for such tasks requires an extensive amount of annotated data, especially in terms of the diversity of traffic signs. The collection of data is both time-consuming and costly. Recent advances in large foundation models such as BERT, DALL-E, and GPT [5] offer a promising solution to this challenge by using knowledge already acquired from these foundation models for such specific downstream tasks [5]. Thus, the need for large additional training data can be minimized, because fine-tuning often requires only a limited amount of data or specific guidance. This study explores the application of pre-trained foundation models using *Meta*’s Segment Anything Model (SAM) [6]. SAM is a transformer-based foundation model developed for semantic segmentation. The use of the transformer model architecture enables more meaningful models to be created in less time through parallel training [5]. A large amount of training data utilized for pre-training the SAM improves the model’s capabilities in the field of computer vision. The primary purpose of this study is to assess whether the SAM can maintain its performance despite being trained on a significantly reduced dataset, thereby lowering the barriers for subtasks and making them more accessible and cost-effective. The performance of the foundation model is evaluated in comparison to a leading architecture in the field of traffic sign segmentation.

The architecture of the SAM consists of an image encoder, prompt encoder, and mask decoder. Its heavyweight image encoder is based on a vision transformer (ViT) [7] architecture and extracts the input image features into image embeddings. The prediction depends on an input that is provided as a prompt. This prompt may take the form of one or more points, a bounding box, a mask, or text. The prompt is processed by the prompt encoder [6]. The lightweight mask decoder is designed for interactive real-time mask prediction. Several studies have modified the model to align with their specific research objectives. These include studies on remote sensing applications [8,9,10], shadows [11,12], food [13], web pages [14], oil spills [15], and camouflaged objects [16]. SAM fine-tuning has a particular focus on medical image segmentation [17,18,19,20,21] due to the limited availability of data in this field. In general, updating all parameters of the SAM is a time-consuming process. Consequently, numerous studies have focused their efforts on parameter-efficient fine-tuning [17]. They employed the use of adapter modules, which are positioned between the encoder transformer layers [11,18]. Subsequently, the adapter, prompt encoder, and mask decoder are fine-tuned on specific data. A significant limitation of the SAM is the dependency of segmentation on prompt input. For this particular task, it is necessary to adapt the SAM for the purpose of automatic traffic sign segmentation. One concept for automatic mask prediction is the use of the automatic mask generation pipeline [6], which employs a large grid of points as a prompt. However, the automatic mask generation pipeline is not suitable for productive use due to its long calculation time. For instance, in the context of SAM automation, there are approaches for developing a prompt generator, which generates prompts for the prompt encoder [10,22,23,24]. Another variant employs learnable embeddings as prompts [24].

In this work, the original SAM image encoder is combined with two distinct decoders to determine whether there is an influence of a decoder of the foundation model, which only contains 1% of all the model parameters. In addition to the original mask decoder (referred to as SAM-Fine-ViT_H_), a decoder containing convolutional layers (referred to as SAM-Conv-ViT_H_) is used. One significant drawback of the powerful SAM encoder is its long processing time, which is a consequence of the considerable number of parameters involved. Consequently, a parameter-distilled encoder version ViT_T_ [25], created based on knowledge distillation to enhance performance, is employed. It is an opportune moment to consider whether the tiny version would yield comparable results, even with limited training data. The heavyweight image encoder has consequently been replaced with the tiny image encoder, while only the decoder has undergone fine-tuning. The decoder also contains convolutional layers (referred to as SAM-Conv-ViT_T_).

Given the importance of traffic sign recognition for autonomous driving systems, as well as urban mobile mapping for maintenance planning or accident prevention, the field is widely covered in the scientific literature. However, in this field, the dominant approach is the use of convolutional neural networks (CNNs) [26,27,28,29,30,31,32]. In contrast to object detection, semantic segmentation of traffic signs is a relatively underrepresented field. References [33,34,35,36,37,38] demonstrate that the Faster R-CNN model is a widely used tool for the detection of traffic signs, with the model achieving highly satisfactory results. In addition to the Faster R-CNN model, its extension, the Mask R-CNN model is used, as it also enables instance segmentation [39,40]. One of the few studies on sign segmentation uses a fully connected network [27]. Another approach [41] employs traffic sign recognition through segmentation with a specially developed SegU-Net, a fully connected network, that represents a combination of Seg-Net [42], and U-Net [43]. In 2022, an initial approach was implemented to investigate the use of ViT for traffic sign recognition [44]. It was found that the tested CNNs outperformed the ViT architecture. However, these were pre-trained compared to the transformer architectures. The publication points out the advantages of the ViT in terms of its shorter training duration. Another paper comparing the feature extraction of CNNs with that of ViT [45] demonstrates that the original ViT models are outperformed in the detection of advanced CNNs. Nevertheless, when a ViT is employed as a backbone model in the context of detection and compared to CNN backbone models, the ViT models demonstrate superior performance [46]. The three models, which are based on the SAM architecture, are evaluated and compared to the Mask R-CNN architecture as a well-established and leading framework for traffic sign segmentation.

In this study, we investigate the adaptability of foundation models to a specific task with minimal additional training data. Our contributions can be summarized as follows:

- A comparative evaluation between a state-of-the-art architecture provides for a specific task and a foundation model, focusing on their performance with reduced training data.
- Two distinct decoders applied to the same large-scale encoder are compared, examining their impact on model performance.
- The effects of knowledge distillation from foundation models are examined in the context of training data reduction.
- The results are evaluated against multiple benchmark datasets, ensuring the robustness, reliability, and generalizability of our findings.
- Model performance is measured based on the intersection over union (IoU), precision–recall, and their segmentation ability on an instance basis.

The main documentation is structured in two sections: Section 2 considers the structures of the four implemented architectures, while Section 3 presents the results of the implementation. In Section 2, the implementation details are provided, including descriptions of the data preprocessing steps, the hyperparameter selection process, and the training setup. The datasets used are described in detail in Section 3, followed by the presentation of segmentation results on different datasets. Additionally, the impact of training data reduction is analyzed, considering the IoU and precision–recall metric and presenting instance-level segmentation results. The results are interpreted in Section 4 and summarized in Section 5.

## 2. Methods

For implementation, the pre-trained ViT_H_ (huge) encoder is employed. The architectural structure enables the encoder to be used independently. During preprocessing, the image is scaled to 1024×1024 pixels and encoded into 64×64 with 256 feature maps [6]. For automated use, the prompt encoder is completely removed, so the prompt tokens that correspond to the output of the prompt encoder are replaced by learnable embeddings. The number of model parameters that are frozen and fine-tuned during training, along with their respective performance, is presented in Table 1. The performance is quantified in terms of floating-point operations per second (FLOPS) using an NVIDIA A100 SXM4 80GB GPU with an input tensor of dimensions 1024 × 1024 × 3.

**Figure 1:**
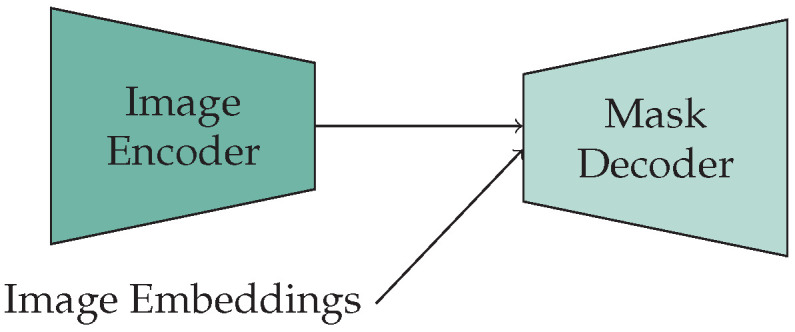
SAM-Fine-ViT: SAM architecture without prompt encoder.

For SAM-Fine-ViT_H_, the originally designed lightweight decoder is utilized (Figure 1). It initially processes the embeddings with a transformer, and subsequently with 2D transposed convolutions, to increase the image size to 256×256. The transposed convolution employs a 2×2 kernel with a stride of two, which doubles the resolution and reduces the number of feature maps to a quarter. After a transposed convolution, layer normalization follows. The output of the up-scaling step is employed after a multilayer perceptron to generate the masks and the IoU token.

**Figure 2:**
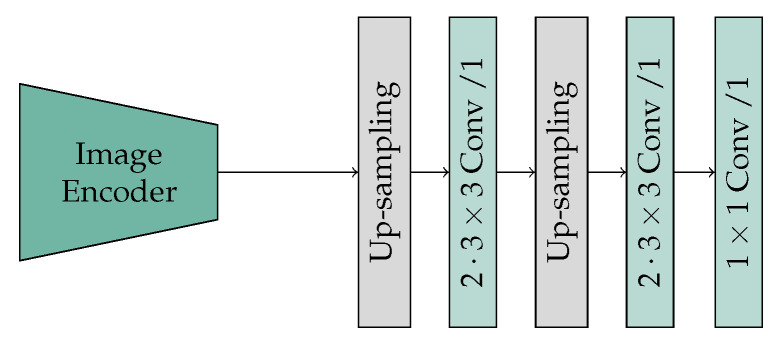
SAM-Conv-ViT_H/T_: SAM image encoder with convolutional decoder.

The decoder of SAM-Conv-ViT corresponds to the final two up-sampling steps, which combine an up-sampling and convolution layer, of the ascending path of the U-Net implementation [43]. The initial step of the ViT patch-embedding process involves cropping image patches to a size of 16 × 16. Consequently, no encoding information is stored, and no skip connections can be used. Following the up-sampling, which doubles the size of the output and halves the number of feature maps, a subsequent double convolution is then applied (Figure 2). A simple parameter-efficient bilinear interpolation is employed for up-sampling. The convolution is performed with a 3×3 kernel, a stride of one, and a zero-padding of one, and it is supplemented by batch normalization and the rectified linear unit (ReLU) activation function. Finally, 64 feature maps with a resolution of 256×256 remain, which are then processed into a binary mask by a 1×1 convolution layer.

**Figure 3:**
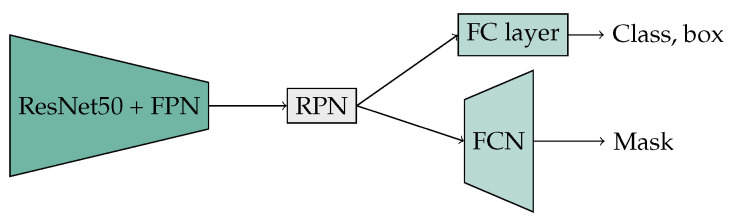
Mask R-CNN architecture.

In accordance with reference [47], the Mask R-CNN implementation (Figure 3) employs a ResNet50 backbone model for feature extraction, extended by a feature pyramid network (FPN) [48]. The FPN employs hierarchical processing of image information at four different resolutions. This approach is advantageous for the recognition of traffic signs, as these are often very small. In ResNet50, residual blocks derived from convolution operations with 1×1, 3×3, and 1×1 kernels with a stride of one were employed. To enhance the extraction of features, a ResNet50 pre-trained on the COCO dataset was utilized. The weights of the initial layer of a ResNet50 are frozen for the training process. This layer is typically responsible for processing fundamental features, such as edges and simple shapes, which are frequently useful in numerous image-recognition tasks. Based on the output of the encoder, a region proposal network (RPN) is used to predict potential bounding boxes, from which smaller feature maps are formed. A small fully convolutional network (FCN) is added to the architecture as a decoder for the pixel-based prediction of the binary mask. Additionally, a fully connected (FC) layer has been included to provide the necessary classification capabilities.

### Implementation Details

The decoders are subjected to fine-tuning on a dataset for traffic sign segmentation, while the encoder parameters (or a subset of them in the case of Mask R-CNN) are held constant throughout the training process. In the preprocessing step, the input images are scaled to the dimensions required by the architectures. Additionally, the red, green, and blue (RGB) values of the images are normalized with the average RGB and standard deviation (SD) values of images used for training the SAM and Mask R-CNN. The SAM encoder architecture requires an image size of 1024×1024, while the output mask of the decoder is 256×256. For Mask R-CNN, an input and output image size of 512×512 is employed.

To enhance the robustness and generalizability of architectures, the utilization of data augmentation for road images in the context of traffic sign recognition has a beneficial impact on the outcomes [38,39]. In this study, a series of functions, including brightness, contrast, rotation, distortion, blurring, and noise, were employed for data augmentation in the context of street scenes. The images have been adjusted to handle different lighting situations, varying camera settings and perspectives, and environmental influences such as motion blurring caused by high travel speeds. For the implementation, the data augmentation functions of torchvision, a library from PyTorch [49], and albumentation [50] are used.

The segmentation models are trained using an equal-weighted combination of the Dice and Binary Cross-Entropy loss functions. This approach is particularly well-suited for data sets with an imbalanced class distribution and for small objects [51]. Additionally, the Adam optimization algorithm [52] is employed. An initial learning rate of 0.1 (for all SAM models) and 0.001 (for Mask R-CNN) is scheduled based on the publication by Loshchilov et al. (2017) [53]. The approach employs the cosine annealing warm restarts function, which periodically repeats the descending part of the cosine function. The training phase was conducted using a maximum of five hundred epochs, but the process was terminated prematurely when the validation metric reached a stable point or began to decline. All models were trained on an NVIDIA A100 SXM4 80 GB GPU with a batch size of eight, which aligns with the maximum available resources of the working memory. The implementation is carried out in Python using the PyTorch [49] framework.

## 3. Results

The IoU metric utilized in the present study is based on the previously published paper on traffic sign instance segmentation and classification with data augmentation by Yoá et al. (2023) [39]. The IoU threshold value categorizes the segmented area as either a true positive (TP), a true negative (TN), a false positive (FP), or a false negative (FN) prediction. The results presented below demonstrate the IoU for a threshold of 0.75 for the class *traffic sign*.

### 3.1. Datasets

**Figure 4:**
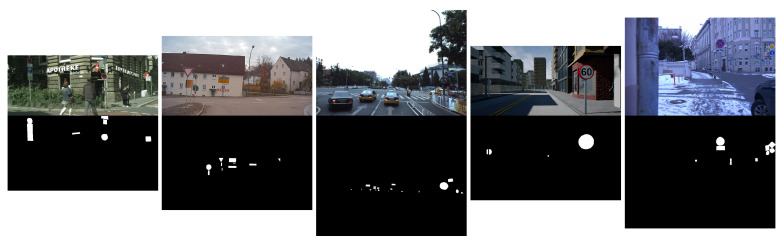
Image and mask examples of the datasets. From left to right: Cityscapes, A2D2, ApolloScape, IDDA, Mapillary Vistas.

The segmentation results of the different architectures are evaluated using five datasets for traffic sign segmentation. A subset of images from each dataset is selected, containing the class *traffic sign*. Figure 4 provides an example of one image and mask per dataset. The size, mean, and SD of the images in Table 2 illustrate the considerable differences between the datasets.

The *Cityscapes Dataset* [54] comprises segmented urban images from 18 German cities, with 5000 images finely annotated and 20,000 images coarsely annotated. Only the finely annotated data are utilized in this study. Of the 5000 images, 3475 annotated images are publicly available. Of these, 94% (3281) contain the class *traffic sign*. The *Audi Autonomous Driving Dataset* (A2D2) [55] comprises 41,277 frames of semantic segmented images of highways, country roads, and cities in the south of Germany. A total of 1841 front–center images were utilized for model testing. A dataset with high diversity is the *Mapillary Vistas Dataset* [56]. The street-level photos were collected using different cameras from a rich community of contributors worldwide. There are 25,000 images within six continents. The official validation subset of 1726 images is used for testing purposes. A significant advantage of the Mapillary Vistas dataset is its annotation of traffic sign instances. A further dataset for autonomous driving is the *ApolloScape Dataset* [57], which contains 146,997 semantically annotated images from four regions in China. A total of 2269 selected examples were used for testing purposes. Furthermore, a large synthetic dataset is available in the form of *IDDA* [58]. This dataset imitates over 1M images of six US towns captured under three different weather conditions. For testing, 3075 images captured during clear daylight are utilized.

### 3.2. Segmentation Results for Different Datasets

To assess the performance of the architectures, the models were tested on five distinct datasets (Section 3.1).

**Figure 5:**
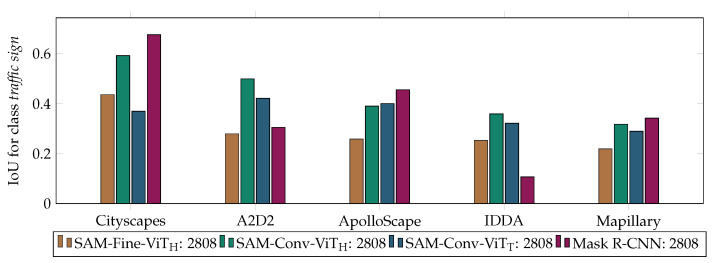
Results of the IoU for the class *traffic sign* for the models trained on the entire dataset (2808 images).

The segmentation results of different models across different test datasets are presented in both tabular form in Table 3 and visually in Figure 5. All four architectures trained on the Cityscapes dataset achieved their respective best results on the same test dataset. The models are particularly well adapted to the characteristics of this specific data set. The Mask R-CNN model achieved its top performance on Cityscapes. In general, the lowest segmentation results were observed on the IDDA test data, which are of a synthetic origin. This was particularly evident in the case of the Mask R-CNN model. Notably, the SAM-Conv-ViT_H_ model exhibits the highest average performance (0.432), indicating that this model generalizes most effectively across the diverse test datasets. Nevertheless, the Mask R-CNN model exhibits the highest values in individual datasets (Cityscapes, ApolloScape, and Mapillary). In comparison with the SAM-Conv-ViT_H_ model, it can be observed that the SAM-Conv-ViT_T_ demonstrates minimal differences when applied to different datasets, but the overall results are lower than those obtained using the SAM-Conv-ViT_H_ model. In overall consideration, the SAM-Fine-ViT_H_ architecture achieved the lowest results.

### 3.3. Reduction of Training Data

**Figure 6:**
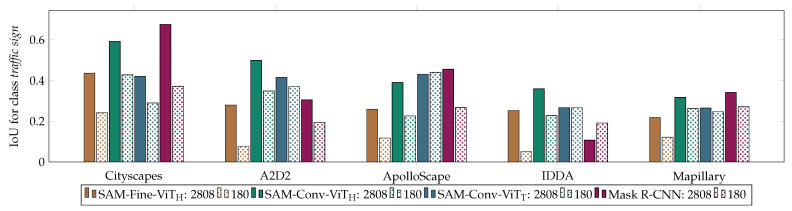
Results of the IoU for the class *traffic sign* for the models trained on the entire dataset (2808 images) and on a smaller subset (180 images).

To evaluate the performance of the segmentation architectures on a limited amount of training data, the training was performed on a subset of 5.5% of the Cityscapes data. The impact of reducing the training dataset to 180 images is illustrated in Table 4, which also presents the difference in the results when training on the full dataset. Figure 6 illustrates the results in a visual format.

Notably, all models exhibited a significant decline in performance when trained on a smaller dataset. The mean accuracy loss (mean diff) was greatest for SAM-Fine-ViT_H_ (−0.1672). It can be observed that the performance of SAM-Fine-ViT_H_ exhibited a pronounced decline as a consequence of the reduction in the quantity of training data. This decline occurs to a greater extent than in the case of SAM-Conv-ViT_H_, which employed the same encoder. The SAM-Conv-ViT_T_ model demonstrates the most effective performance when trained on a reduced training set and is the most robust to the reduction in training data, exhibiting minimal impacts. The reduction in the accuracy of SAM-Conv-ViT_H_ due to the reduction in the training data is greater than that of Mask R-CNN. However, the greatest loss of accuracy is observed on the Cityscapes dataset, particularly for Mask R-CNN.

**Figure 7:**
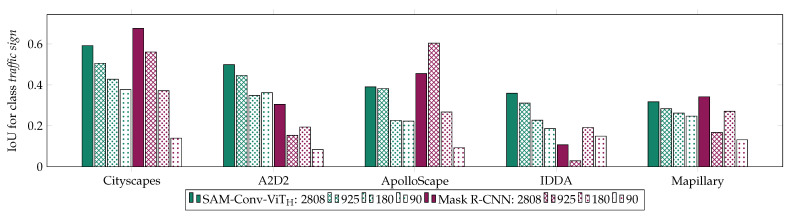
Results of the IoU for the class *traffic sign* for SAM-Conv-ViT_H_ and Mask R-CNN trained on the entire dataset (2808 images) and on smaller subsets (925, 180, 90 images).

For further analysis, the SAM-Conv-ViT_H_ and Mask R-CNN were trained on 925 (32.9% of the total) and 90 (3.2% of the total) Cityscapes images (Figure 7). The smaller subsets comprise images of the larger ones. As expected, the two models generally show a decline in accuracy with the reduction of data. Mask R-CNN exhibits a more pronounced decline in accuracy when confronted with limited training data, whereas SAM-Conv-ViT_H_ demonstrates a relatively stable and foreseeable reduction in accuracy. For very limited training data (90 images), the foundation model SAM-Conv-ViT_H_ outperforms Mask R-CNN, SAM-Conv-ViT_H_ has a decrease in IoU of −0.15, and Mask R-CNN has a decrease of −0.26 over all datasets, referring to the models trained on the entire dataset.

### 3.4. Precision–Recall

**Figure 8:**
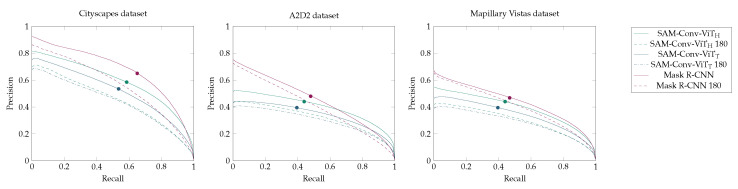
Precision–recall curve. The point where recall and precision are equal is marked.

The precision–recall curve is employed as a metric to assess the segmentation outcomes across a range of threshold values (Figure 8). The observation is considered for the three datasets, Cityscapes, A2D2, and Mapillary Vistas, as the ground truth does not include annotations for the backsides of the signs, which affects the accuracy results.

As anticipated, the precision of all the models is observed to decline as recall increases. Mask R-CNN achieves the highest precision values relative to recall values for all three datasets, except for the high recall values observed in the A2D2 dataset. The graphs also demonstrate that SAM-Conv-ViT_H_ achieves better results than SAM-Conv-ViT_T_. The performance is quantified based on the area under the curve. The smallest reduction in precision was exhibited by SAM-Conv-ViT_T_ (by −0.065) and Mask R-CNN (by −0.601) as a consequence of the decrease in the training data. In contrast, SAM-Conv-ViT_H_ (by −0.105) displayed the greatest reduction. For lower thresholds, the discrepancies in precision between the datasets are more noticeable.

### 3.5. Sign Instances

Due to the varying sizes of the traffic signs, the segmentation results are considered for each sign instance separately. Therefore, the 9277 specific signs of the Mapillary Vistas Dataset (Section 3.1) were divided into five size categories (XS, S, M, L, XL) based on their pixel areas (Table 5). The number of detected signs with IoU values greater than 0.0 for a threshold of 0.75, irrespective of the segmentation results, is counted (det. ratio), and the IoU of these is calculated (IoU of det. signs). For instance-based results, the IoU is calculated using only the pixels in a bounding box around the specific sign instance. The bounding box is enlarged to about half the size of the actual sign. It should be noted that FP outside the bounding boxes will not be considered.

When analyzing the segmentation results based on individual instances, it can be observed that only a relatively small number of signs are identified. The result indicates that, on average, only 32.04% of all sign instances were identified by SAM-Conv-ViT_H_, 20.43% were identified by SAM-Conv-ViT_T_ and 22.60% were identified by Mask R-CNN. Both the quantity (det. ratio) and the quality (IoU of det. signs) of recognized signs increase as the size of the objects increases (Table 6). This trend is recognized across all three models. It is important to note that the SAM-Conv-ViT_H_ model consistently exhibits higher detection rates compared to the other two models across all size categories. Mask R-CNN demonstrates robust performance in terms of IoU, particularly in the L and XL categories. This performance was significantly better than the other two models. SAM-Conv-ViT_H_ and SAM-Conv-ViT_T_ have the strength to identify a greater number of signs, while Mask R-CNN is more adept at segmentation.

The reduction in training data has a minimal impact on the detection ratio and IoU of SAM-Conv-ViT_T_ (Table 6). In the case of a high detection rate, SAM-Conv-ViT_T_ and SAM-Conv-ViT_H_ demonstrated superior performance. SAM-Conv-ViT_H_ achieved the highest detection rate for the L and XL categories. The reduction in the Mask R-CNN detection ratio (by −17.0% for the XL category) is comparable to that of SAM-Conv-ViT_H_ (−15.2% for the XL category). Notably, that the segmentation strength (as measured by IoU) of Mask R-CNN is not affected by the reduction in training data, in contrast to SAM-Conv-ViT_H_.

## 4. Discussion

The segmentation of traffic signs is challenging due to their variability in shape, color, and small size. Across different model architectures, the number of detected signs is low. The smaller the size of the traffic signs, the lower the recognition rate. Among the models evaluated, the one with the greatest number of parameters, SAM-Conv-ViT_H_, exhibited the highest overall performance, followed by Mask R-CNN and SAM-Conv-ViT_T_. As expected, all the models demonstrated the highest prediction accuracy on the Cityscapes dataset due to its use for optimization. Mask R-CNN, which serves as the benchmark leading model, achieved the best results on the Cityscapes dataset. However, there are limitations to its adaptability to different datasets. In contrast, SAM-Conv-ViT_H_ outperforms Mask R-CNN in accuracy across all datasets. Similarly, SAM-Conv-ViT_T_ delivers less-impressive results overall but demonstrates the most consistent performance across different datasets. The models with a robust encoder indicate superior generalization capabilities. The analysis shows that SAM-Conv-ViT_H_ demonstrates better segmentation accuracy than SAM-Fine-ViT_H_. It can be concluded that the decoder of the foundation model has a notable impact, despite comprising only 1% of the parameters. The SAM-Fine decoder was initially developed as a lightweight decoder to enable real-time mask prediction based on a prompt. However, it became evident that this decoder was not suitable for the task of traffic sign segmentation.

With limited training data, Mask R-CNN, behind SAM-Fine-ViT_H_, exhibits the most pronounced performance reduction. The results of the training data reduction indicate that SAM-Conv-ViT_H_ demonstrates better robustness to smaller training datasets compared to Mask R-CNN. This is due to the greater number of pre-trained parameters, which are more relevant when training data are limited. SAM-Conv-ViT_T_ benefits from knowledge distillation, showing superior generalization capabilities even with fewer parameters and demonstrating no decrease in accuracy when the training data are reduced. However, the precision–recall observation demonstrates noteworthy outcomes. Mask R-CNN exhibits a minimal reduction over the entire threshold spectrum. Contrary to expectations, SAM-Conv-ViT_H_ shows the highest reduction over the precision–recall curve, suggesting that extensive pre-training is not essential for this task. The curve observation is more meaningful than considering a single IoU threshold at 0.75.

**Figure A1:**
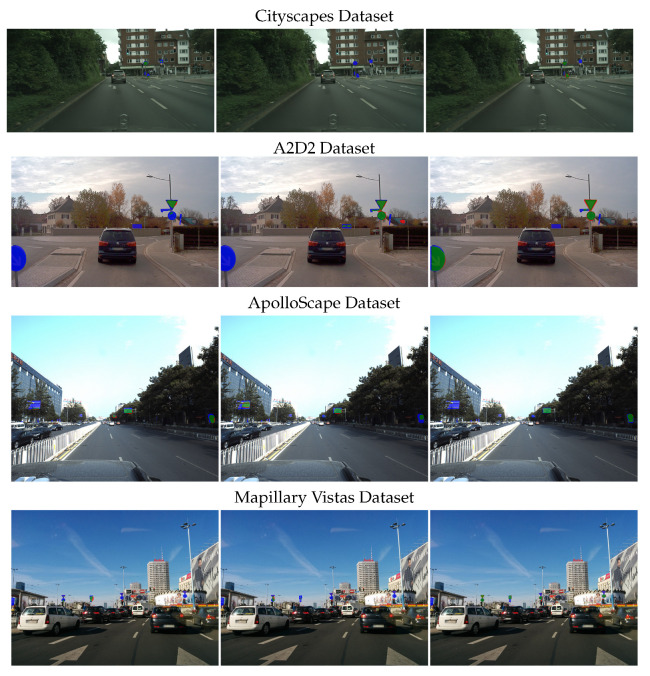
Examples of mask predictions for class *traffic sign* (green: TP, red: FP, blue: FN). From left to right: SAM-Conv-ViT_H_, SAM-Conv-ViT_T_, Mask R-CNN.

Instance-level observations indicate that SAM-Conv-ViT_H_ is more effective at identifying sign instances, whereas the Mask R-CNN model demonstrates superior performance in terms of correct segmentation. A correlation is identified between the encoder and the quantity of recognized masks, as well as between the decoder and the quality of the masks. Although SAM-Conv-ViT_T_ once again underperformed compared to SAM-Conv-ViT_H_ and Mask R-CNN when trained on the entire dataset, it demonstrated a clear advantage in terms of data reduction, exhibiting no decrease in accuracy. The main issue with the foundation model, as with the others, is the low detection rate, particularly for smaller signs. The process of down-sampling employed by the encoder resulted in the loss of certain image details, particularly those of a smaller scale. The effective performance of Mask R-CNN can be explained using an FPN, which processes images at varying resolutions and predicts sharp-edged masks without interpolation. In contrast, SAM-Conv-ViT_H_ relies on interpolation, leading to curved edges in the segmentation masks (Figure A1). The effects of interpolation on the quality of mask prediction are particularly pronounced in the case of small objects, such as traffic signs. To improve the segmentation ability of the SAM, a high-quality version of the SAM, HQ-SAM [59], has been developed. However, HQ-SAM does not outperform the original SAM in the case of small objects measuring less than 1024 pixels. For the Cityscapes dataset, for example, this means approximately 67% of the traffic signs fall into this category. Consequently, this area was not investigated further in this study.

## 5. Conclusions

The segmentation results from both the entire dataset and smaller datasets indicate that the foundation model does not clearly outperform the leading model, Mask R-CNN, in the specific field of traffic sign segmentation. This is particularly relevant when considering the drawbacks associated with the collection and annotation of extensive amounts of training data, as well as the lengthy training and processing times involved. To reduce the need for training data, our findings indicate that the knowledge-distilled model produces the most stable results. These impressive achievements demonstrate that quality is more important than quantity. There is significant redundant information in the dataset that does not advance the models. This example shows that more parameters or training data do not necessarily lead to better results. The main task is to obtain those with the most influence, with the aim of creating a generalized effect. However, applying knowledge distillation techniques to foundation models, which reduces the number of parameters and computational costs, is only applicable if a teacher model for knowledge distillation has already been established.

## Figures and Tables

**Table 1:** The number of model parameters and the performance of the models, measured in FLOPS.

Model	All	Frozen	Fine-Tuned	FLOPS in 1/s
SAM-Fine-ViT_H_	640,803,746	637,026,048	3,777,698	218.0
SAM-Conv-ViT_H_	644,956,546	637,026,048	7,930,498	217.8
SAM-Conv-ViT_T_	13,996,030	6,065,532	7,930,498	2413.8
Mask R-CNN	43,922,395	222,400	43,699,995	1439.6

**Table 2:** The number of available images per dataset, along with their image sizes, means, and SDs of RGB.

Dataset	Training	Testing	Size	Mean [r, g, b]	SD [r, g, b]
Cityscapes	2808	473	2048×1024	[73.6, 83.3, 72.9]	[45.3, 46.4, 45.6]
A2D2	-	1841	1920×1208	[129.4, 121.6, 119.2]	[47.6, 54.0, 60.1]
ApolloScape	-	2269	3384×2710	[125.7, 135.3, 141.0]	[94.3, 97.6, 97.2]
IDDA	-	3075	1920×1080	[90.7, 95.0, 103.8]	[50.6, 51.8, 57.4]
Mapillary Vistas	-	1726	arbitrary	[107.7, 117.7, 120.8]	[62.6, 65.9, 72.5]

**Table 3:** Results of the IoU for the class *traffic sign* for the models trained on the entire dataset (2808 images). For each dataset, the highest IoU is highlighted.

Dataset	SAM-Fine-ViT_H_	SAM-Conv-ViT_H_	SAM-Conv-ViT_T_	Mask R-CNN
Cityscapes	0.436	0.592	0.370	**0.676**
A2D2	0.279	**0.499**	0.422	0.305
ApolloScape	0.259	0.390	0.401	**0.456**
IDDA	0.253	**0.360**	0.322	0.107
Mapillary Vistas	0.219	0.318	0.290	**0.342**
Mean	0.289	**0.432**	0.361	0.377
SD	0.086	0.098	**0.050**	0.195

**Table 4:** Results of the IoU for the class *traffic sign* for the models trained on a smaller subset of data (180 images). The differences relate to the models that are trained on the entire dataset (2808 images). For each dataset, the highest IoU and the lowest difference are highlighted.

	SAM-Fine-ViT_H_		SAM-Conv-ViT_H_		SAM-Conv-ViT_T_		Mask R-CNN	
Dataset	Small	Diff	Small	Diff	Small	Diff	Small	Diff
Cityscapes	0.242	−0.194	**0.428**	−0.164	0.290	**−0.080**	0.372	−0.304
A2D2	0.078	−0.201	0.349	−0.150	**0.370**	**−0.052**	0.194	−0.111
ApolloScape	0.118	−0.141	0.226	−0.164	**0.440**	**+0.039**	0.268	−0.188
IDDA	0.050	−0.203	0.228	−0.132	**0.267**	**−0.055**	0.192	+0.085
Mapillary Vistas	0.122	−0.097	0.263	−0.055	0.247	**−0.043**	**0.271**	−0.071
Mean	0.122	−0.167	0.299	−0.133	**0.323**	**−0.038**	0.259	−0.118

**Table 5:** Definition of instance sizes.

Size Category	Pixel Area A	No. of Instances
XS	1 ≤ A ≤ 31	1857
S	32 ≤ A ≤ 73	1854
M	74 ≤ A ≤ 179	1863
L	180 ≤ A ≤ 521	1850
XL	522 ≤ A ≤ 74,343	1853

**Table 6:** Results based on instance size: the number of detected signs with IoU values greater than 0.0 is counted (det. ratio), and the IoU of these is calculated (IoU of det. signs). For each size category, the highest det. ratio and the highest IoU of det. signs are highlighted.

	**SAM-Conv-ViT_H_**		**SAM-Conv-ViT_T_**		**Mask R-CNN**	
**Size Category**	Det. ratio	IoU of det. signs	Det. ratio	IoU of det. signs	Det. ratio	IoU of det. signs
XS	** 0.92%**	0.051	0.32%	0.055	0.81%	**0.060**
S	** 3.83%**	0.089	1.08%	0.051	3.18%	**0.093**
M	**16.64%**	0.176	3.76%	0.102	12.18%	**0.270**
L	**53.51%**	0.323	26.59%	0.261	37.19%	**0.518**
XL	**85.48%**	0.522	75.61%	0.474	59.79%	**0.674**
	**SAM-Conv-ViT_H_ 180**		**SAM-Conv-ViT_T_ 180**		**Mask R-CNN 180**	
**Size Category**	Det. ratio	IoU of det. signs	Det. ratio	IoU of det. signs	Det. ratio	IoU of det. signs
XS	0.22%	0.032	0.27%	0.030	** 0.43%**	**0.063**
S	0.65%	0.050	0.32%	0.058	** 1.46%**	**0.173**
M	3.11%	0.130	2.09%	0.111	** 5.53%**	**0.388**
L	**24.05%**	0.229	14.92%	0.212	16.92%	**0.515**
XL	**70.26%**	0.398	67.40%	0.432	42.80%	**0.684**

## Data Availability

No new data have been created.

## Abbreviations

The following abbreviations are used in this manuscript:

CNN	convolutional neural network
FC	fully connected
FCN	fully convolutional network
FN	false negative
FP	false positive
FPN	feature pyramid network
IoU	intersection over union
ReLU	rectified linear unit
RGB	red, green, and blue
RPN	region proposal network
SAM	segment anything model
SD	standard deviation
TN	true negative
TP	true positive
ViT	vision transformer
